# Mr. Smyth, on Flexible Metallic Bougies & Catheters

**Published:** 1807-05

**Authors:** W. Smyth

**Affiliations:** Tavistock Street, Covent Garden


### *To the Editors of the Medical and Physical Journal.*

GENTLEMEN,

IT is now some years, since I had the satisfaction of announcing through the medium of your useful publication, an important discovery to the medical profession, on that part of surgery which relates to the composition and application of bougies. This discovery, from the materials of which it is composed, is termed the flexible metallic bougie. On making my invention known, I had the pleasure sure of soon finding that it received from the most intelligent characters, such approbation, as I flattered myself it was entitled to from a long tried experience in my own practice; but like every other new invention, it received from others an unqualified censure, without their having given themselves the trouble of a proper examination, and condemn it on their own theoretical ideas alone. Since that time, however, it gives me no small pleasure to observe, that many of its then opposers are now become its warmest advocates; and that they have liberally and candidly withdrawn the hasty and ill timed opinion they then delivered concerning it.

At the same time, it would be ungrateful, were I not here to express in a particular manner, my obligation to that eminent and distinguished surgeon, Mr. Birch, who, on first seeing the instrument, gave it his full approbation, and from a conviction of its utility, encouraged me to proceed in laying it before the faculty and the public.—Nor are my thanks less due to the Hospital Surgeons of the metropolis in general, who, with much candor, employed it on my application for the purpose of investigating its merits, and have given me the most valuable and handsome testimonies of its utility, both in their public and private practice.

My present object therefore is, to offer some remarks on its improved state, and to shew that, like every other useful invention, its fate has been to acquire with time an increasing reputation that is daily making progress, and that it is not of that trifling or uncertain benefit, which renders it necessary to crave public notice from party enthusiasm or high individual character.

Even from the opposers of this improvement, I confess I have received several useful hints, which have rendered it more complete. At first, the composition of the metal was formed rather soft, with the view of making it come as near in its texture to the pliancy of the plaster bougie as possible, and that the metallic bougie might owe its superiority more to its smoothness of surface than to any other circumstance. But a variety of trials soon convinced me that the pliancy of the plaster bougie was no recommendation, and that this fault required to be corrected, whatever the composition was, that a certain firmness was necessary to give a due resistance; and without this firmness being sufficient, any instrument of this kind would not answer the purpose for which it was intended. The texture of the metallic bougie was therefore changed, and a greater degree of hardness given to the metal, which produced the additional advantage of enabling it to receive a higher polish and a greater smoothness of surface, so that by these changes I conceive the composition of the metal has received all the requisite properties for being made into an instrument of this kind, which is to pass through a surface delicate and irritable, full of small sinuosities, and which gives a certain resistance to its passage.

The advantages of this smoothness of surface I cannot too strongly dwell upon. I have never passed a plaster bougie, whatever was its size, without giving the patient much pain. The reverse happens with the metallic bougie; it slides along the urethra as it were imperceptibly, and the patients themselves, who have used both, have spoken in the most flattering terms of the different feelings they experience under the use of the one from what they felt with the other. Whether there is any thing peculiar in the metal to occasion this connection with galvanic principles I cannot pretend to say, but every patient, accustomed to the use of bougies, and who has been subject to strictures that often required their use, has uniformly made the same remark to me.

In respect to the size of the metallic bougie, I carefully carried it no further than to what I termed No. 12; a size I thought sufficient for any purpose, being about the average size of the adult urethra in a state of health; but from the suggestions of several surgeons of eminence, I have been induced to make the size still larger, and to carry it as far, in the same proportion, as to No. 16. I know it is the practice of many surgeons, particularly those of the London Hospitals, to make their catheters and other instruments of this kind very large; and there is little doubt that a large instrument, by dilating a greater extent of surface at once, will be easier for the patient than a small one. But in the application of the larger bougies some attention is necessary to be paid to the orifice of the urethra; and the size of the bougie at the point, in all cases, should be accommodated to it at first, whatever the proportion of the instrument may be afterwards. Indeed, where a larger size than No. 12 is employed, the size of it at the point should not exceed that of No. 12, at its thickest end. Nature intends that the orifice of the urethra, like that of most passages, should be more contracted than the diameter of its after course. This takes place for the best purpose, in order that the urine urine may be discharged with greater force, and be thrown to a proper distance. The large bougies therefore I have caused to be made, so as to be no larger in any part than the thick end of No. 12, which facilitates their introduction, and prevents the orifice from being forcibly extended beyond its natural size. But whatever may be the opinion of Surgeons with respect to the length that the size of the bougies may be carried, I only mean to show, that on this point I have endeavoured to form them of all sizes, so as to meet their views.

Independent of these advantages as bougies only, the present instruments are still of equal, if not greater, importance in their form as catheters. The peculiar property they possess when introduced, of lying in the bladder without giving any pain or uneasiness, and without acquiring any offensive incrustation, or suffering a solution which might be injurious to the patient, gives them a decided preference, sufficient to make them stand high in professional estimation.

Mr. Cline, Surgeon to St. Thomas's Hospital, in his Lectures on Surgery, has recommended my catheter with a screw stopper, to be introduced in those cases that require the water frequently to be drawn off. He observes, "The advantages of this catheter are, that it adapts itself so accurately to the urethra, as to retain its situation without the aid of bandage or ligatures, and that the patient can draw off the water himself, at all times, by unscrewing the stopper." But, although the catheter has been suffered to remain in the bladder upwards of twenty weeks, and only taken out occasionally, without any inconvenience arising from it, yet it would be prudent to withdraw and examine it once a week, lest, by the unavoidable motion of the body, the instrument should become disposed to crack; and, in that case, to introduce another in its stead, of the same size, or a little larger.

Women are not so subject to strictures in the urethra as men, but retention of urine during pregnancy, and after delivery, is very common. The delicacy of some females, however, upon these occasions is such, that they would suffer any pain, even at the risk of their lives, rather than permit the water to be drawn off for them. In order to remedy this inconvenience, I have invented a catheter for their use, and so contrived it as to be carried in the pocket, by which they may at all times relieve themselves with perfect ease and safety, without the assistance of an accoucheur.

It is well known also, that accidents in child-bed, such as laceration of the parts concerned in parturition from the violence of labour, are attended with the most serious evils in bringing on suppression of urine, for which this instrument (with a screw stopper) is so happily adapted; for wherever the laceration is, by this improvement the urine will be prevented from insinuating itself into the cellular membrane; and thus getting into the vagina, in consequence of the catheter constantly filling up the passage of the urethra, which no former instrument of this kind has ever been able to do. It may be retained with the T bandage, and rendered very convenient by letting the screw pass through it by a common slit; but the facility and ease with which the catheters are both introduced and retained in the bladder is so fully established, that the introduction of cases to confirm it here, many of which might be produced, would be unnecessary.

These are a few hints, which I am anxious to communicate to the medical profession, on the superiority of the improvements I have made in this branch of Surgery.—The flexible metallic bougies and catheters have received the unqualified sanction of the first names in practice; and it will give me much satisfaction to receive any communication from the Faculty at large, which may either lead to support what is stated, or to add further to the utility of the invention.

I am, &c.

W. SMYTH.

*Tavistock Street, Covent Garden, April* 12, 1807.

